# Chronic lymphocytic leukemia followed by myelodysplastic syndrome and erythroleukemia

**Published:** 2012

**Authors:** O Cazaceanu, A Iova, AM Vladareanu, M Onisai, C Enache, E Andrus

**Affiliations:** Department of Hematology, Emergency University Hospital Bucharest, “Carol Davila” University of Medicine and Pharmacy, Bucharest

**Keywords:** myelodysplastic syndromes, chronic lymphocytic leukemia, erythroleukemia, second malignant neoplasms

## Abstract

CLL patients are more exposed to develop a second neoplasm, but the association. CLL-MDS is an unusual one. We present the case of a 61-year-old male patient, diagnosed with chronic lymphocytic leukemia in 2007, who developed myelodysplastic features three years later and then acute myeloid leukemia. At diagnosis, the blood tests showed leucocytosis, with lymphocytosis in the peripheral blood and bone marrow. Due to the negative prognostic factors, the patient received treatment with an alkylating agent (FC protocol) and then with alemtuzumab. Three years after being diagnosed with CLL, the patient presented with malaise, recent faintness and fever, with severe anemia and thrombocytopenia. The results from the bone marrow aspirate and biopsy established a new diagnosis: myelodysplastic syndrome. The patient’s general condition was rapidly deteriorating and just two months later, he evolved into acute myeloid leukemia, subtype 6, a very rare type of AML. Soon after, neurological alterations led to cerebral hemorrhage and death. A review of literature is also presented.

Patients with chronic lymphocytic leukemia (CLL) have a higher frequency of second malignant neoplasms. Most reported cases have been therapy-related [**1**]. We present the case of a 61-year-old male patient, diagnosed with chronic lymphocytic leukemia in 2007, who developed myelodysplastic features three years later and then acute myeloid leukemia.

In March 2007, he presented in our clinic with: leucocytosis (WBC ~ 20,000/ μl) and lymphocytosis.

The clinical examination revealed a patient with stable vital signs, normal cardiovascular examination, without lymphadenopathy or splenomegaly. The patient complained of night sweats, extreme fatigue and weight loss in the last six month.

**Laboratory tests:**

The blood tests showed increased WBC-25,600/μl, normochromic normocytic anemia (Hb 11.2g/dl, Ht 32%), low platelet count (122,000/μl). Serum proteins electrophoresis was in normal limits; the Coombs tests were negative. The serological tests for HBV, HCV and HIV were also negative.

The peripheral blood smear showed 82% lymphocytes and smudge cells (**Fig. 1**).

**Fig. 1:**
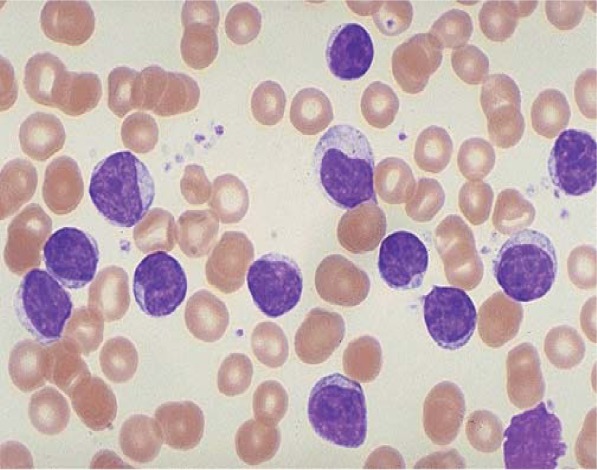
Lymphocytes and smudge cells

The examination of the bone marrow smear revealed 84% monomorphic small lymphocytes. We performed immunophenotyping on peripheral blood, which showed 70% atypical lymphocytes CD19+, CD20+, CD5+, CD23+,CD79b low+, CD38-,FMC7-, ZAP70+ (**Fig. 2-3**).

**Fig. 2-3:**
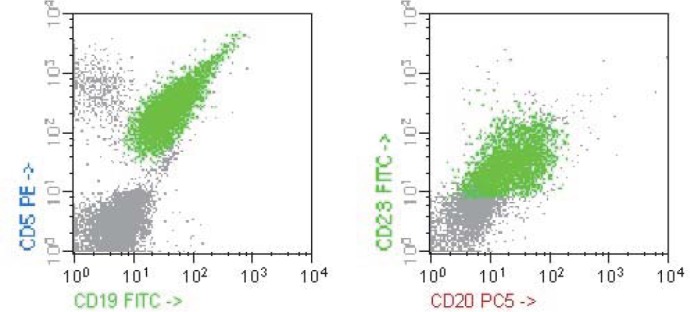
Immunophenotyping peripheral blood; FACS –Calibur acquisiyion, CellQuest software.

The clinical examination and CT scan did not reveal splenomegaly, abdominal adenopathy or lymphadenopathy. The cytogenetic exam could not be performed at that moment.

According to these parameters, the patient was diagnosed with ***CLL stage 0-1***(RAI classification system for CLL).

**Treatment options**

It is important to report that a few months before the arrival of this patient in our clinic, he had a routine blood count, which indicated: normal leucocytes count (WBC ~ 10,000/ μl) and 40% lymphocytes; normal level of hemoglobin and platelets count.

Due to the negative prognostic factors: peripheral lymphocyte doubling time of <12 months, the positive expression of ZAP-70 and the B-symptoms (night sweats, extreme fatigue and weight loss in the last six month), the patient received treatment with an alkylating agent, for almost an year, but the number of the leukocytes remained elevated, more than 20,000WBC/ μl.

From January 2008, the patient had received treatment with FC protocol, with a slowly decrease of the leukocytes number. In October 2008, the patient had normal levels for hemoglobin, platelets and WBC, but with 53% clonal lymphocytes in the peripheral blood and 40% in bone marrow aspirate. For one year, this patient had a linear evolution, without any treatment, until December 2009 when, during a formal evaluation, we discovered an elevated number of WBC, more then 20,000/ μl. Due to the progressive disease we decided to reintroduce the treatment, and the patient received 3 doses of fludara/cyclophosphamide and dexamethasone, without a significant response of the WBC, but with an important decrease of the platelets number.

We decided that the patient had a fludarabine-resistant disease and therefore he was treated with alemtuzumab. This new drug was well tolerated for almost 8 weeks, but then the number of WBC decreased over 2,000/ μl and we decided to stop it.

In September 2010 the patient presented with malaise, recent faintness and fever of >100.5°F. The CBC showed leucocytes of ~ 4.700/μl, moderately severe anemia (Hb ~ 7.9g/dl), and severe thrombocytopenia (PLT ~ 45,000/ μl)

Hematologic reassessment is performed, including bone marrow aspirate and biopsy. The bone marrow aspirate showed: hypercellularity, 5% myeloblasts, *neutrophils with hypogranularity and Pelger-Huet abnormalities*, 60% erythroblasts with megaloblastic and iron deficiency features, with basophilic stippling and Jolly bodies and normal percent of lymphocytes (**Fig. 4-7**).

**Fig. 4:**
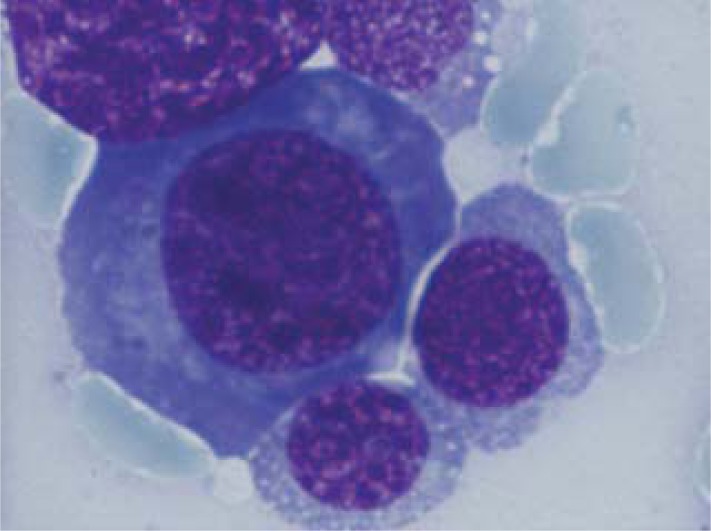
Erythroblasts with megaloblasatic features

**Fig. 5:**
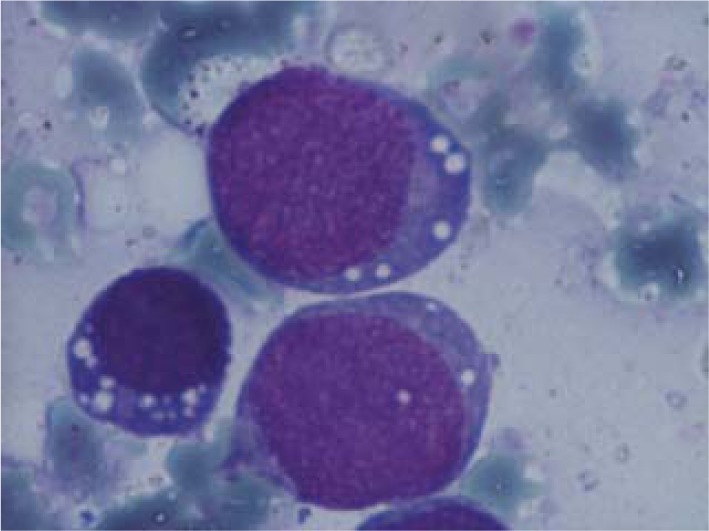
Myeloblasts

**Fig. 6:**
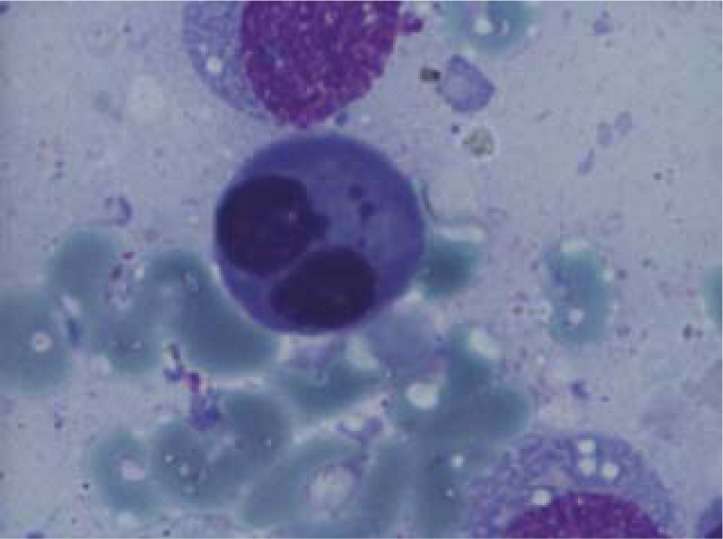
Erythroblast with Jolly bodies

**Fig. 7:**
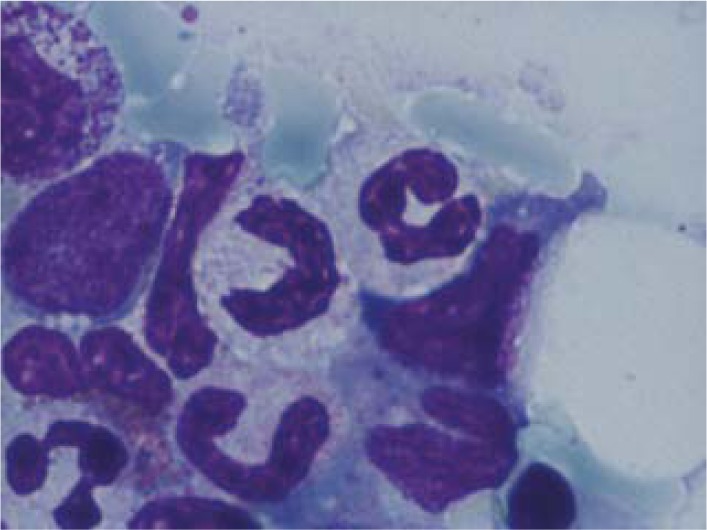
Neutrophils with hyp ogranularity and Pelger-Huet abnormalities

The patient was diagnosed with a second hematologic disease: ***myelodysplastic syndrome with multilineage dysplasia***, according to the result of the bone marrow biopsy. Based on his age (>60 years old) and thrombocytopenia, the patient received supportive therapy, with partial improvement of hematological parameters, but keeping thrombocytopenia. Azacytidine protocol was administered, but the platelet count remained below 20,000/mmc. We administrated also hemostatic treatment, gastric protection and uricosuric.

The patient’s general condition was rapidly deteriorating and at his next evaluation, just two months later, the CBC showed pancytopenia (WBC ~ 2,600/ μl; PLT ~ 10,000/ μl and Hb ~ 10.1g/dl). The peripheral blood smear showed: 30% myeloblasts, 6% neutrophils and 60% lymphocytes. The bone marrow aspirate revealed *33% myeloblasts*, *23% erythroblasts* and *40% lymphocytes* (**Fig. 12-13**); 30% of the erythroblasts were PAS positive (**Fig. 14-15**); the flow cytometry from bone marrow aspirate showed: 25% myeloblasts CD13+ CD 34+ CD117+ and 20% erythroblasts CD61+ Glycophorin A+ CD45 low + (**Fig. 8-11**). The final diagnosis was acute myeloid leukemia subtype 6 (FAB M6).

**Fig. 8-11:**
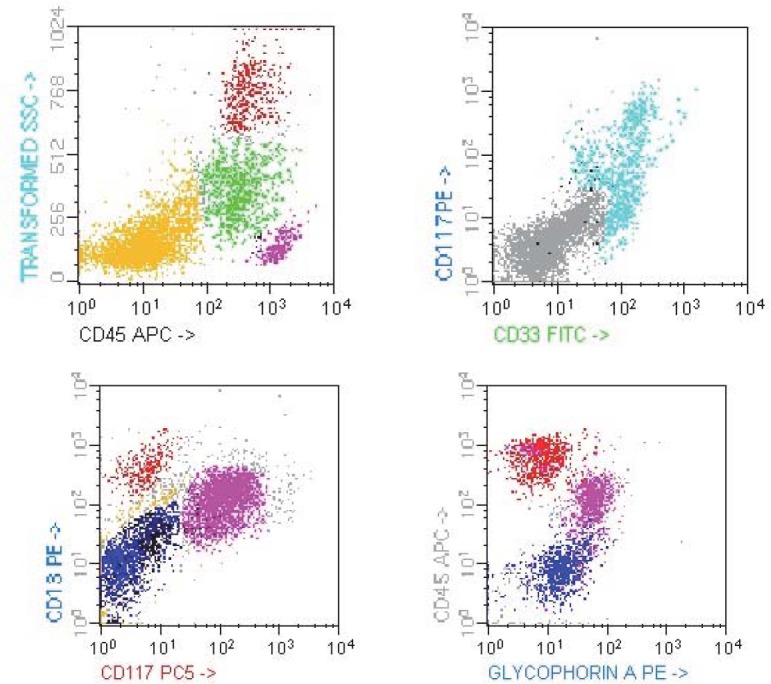
Immunophenotyping the bone marrow; FACS –Calibur acquisiyion, CellQuest software

**Fig. 12:**
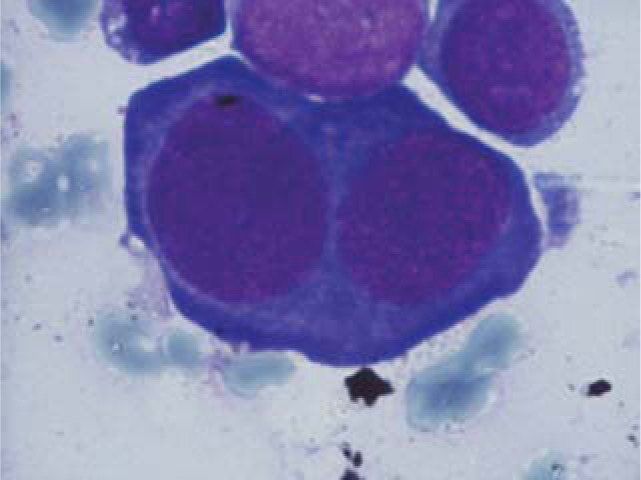
Erythroblasts with two nuclei

**Fig. 13:**
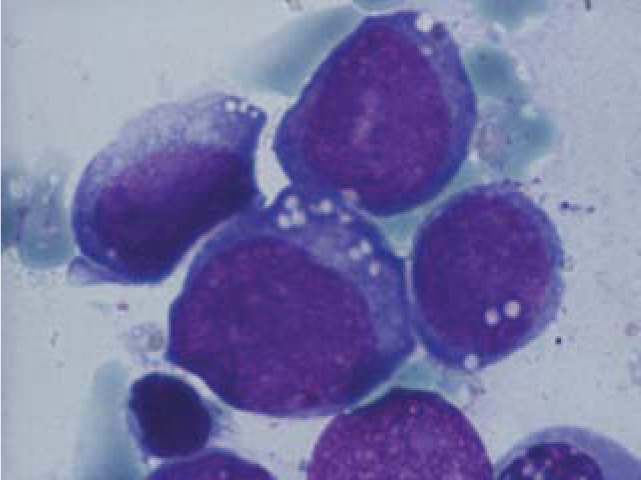
Myeloblasts

**Fig. 14-15:**
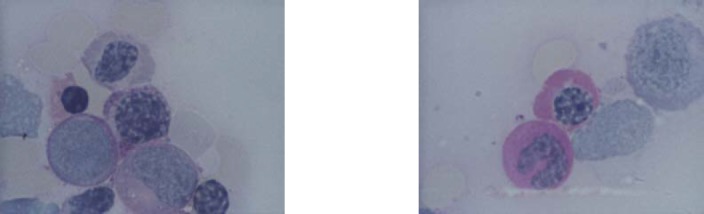
Erythroblasts PAS positive

After being diagnosed with acute leukemia, the patient received specific treatment: chemotherapy - Cytosar - with partial hematologic response.

Both the disease and chemotherapy are responsible for the complications:

a. The patient developed recurrent infections and received injectable antibiotics, antivirals, antifungals and he also required rebalancing treatment (hydro-electrolyte, albumin).
b. Due to severe and persistent thrombocytopenia, the patient developed recurrent episodes of anterior nosebleeds bilateral, requiring nasal tamponade.
c. He installed pain in the right thigh, irradiated in the fingers II, III of the right leg, with difficulty walking. He was neurologically evaluated - possible involvement of L5 in the context disease; he was strongly recommended corticosteroids (low dose due to immunosuppression), with partial improvement of symptoms.

The patient's general status deteriorated progressively, despite intensive supportive therapy. Neurological alteration led to cerebral hemorrhage and death.

**Literature data:** It is known that CLL patients are more exposed to develop a second neoplasm, especialy after being treated with alkylating agents (5 to 9 years) [**1**,**2**].

The t-MDS/AML was first reported after the combination of chemotherapy and radiotherapy for malignant lymphoma and multiple myeloma [**3**]. Prognosis is poor, with a median survival of 7 to 10 months in t-MDS/ t-AML [**1**]. We diagnosed our patient with AML type M6 (erythroleukemia), according to FAB classification. The median age of EL is in the range of 60 to 70 years, approximately one half of cases are therapy-related and have a poor prognosis [**1**]. These findings applied in our case and the patient survived only one month after being diagnosed with AML.

The World Health Organization (WHO) publications and NCI-WG criteria suggest a number of disease activity markers for CLL such as b2-microglobulin, CD23, rapid lymphocyte doubling time, and serum thymidine kinase, for use in predicting patient outcomes [**4**,**5**]. Also, IgVH mutational status, chromosomal aberrations, and expression of zeta- chain–associated protein kinase 70 (ZAP-70) and CD38 are important parameters that complement the conventional prognostic factors previously described [**6**].

High *ZAP-70* expression identified a clinically progressive form of CLL [**5**]. In an investigation evaluating ZAP-70 expression as a surrogate for IgVH mutation status, ZAP-70 expression correctly identified 91% of patients with unmutated IgVH genes, and no patient with mutated IgVH genes overexpressed ZAP-70 [**7**]. The presence or absence of somatic mutations in the expressed immunoglobulin heavy chain variable regions *(IgVH)* of chronic lymphocytic leukemia (CLL) cells provides prognostic information. Patients whose leukemic cells express unmutated *IgVH* regions (Ig-unmutated CLL) often have progressive disease, whereas patients whose leukemic cells express mutated *IgVH* regions (Ig-mutated CLL) more often have an indolent disease [**8**,**9**].

**In evolution**, CLL may transform to a malignant lymphoid disorder or to a solid neoplasm [**10**]. Richter transformation refers to clonal evolution, because it has been demonstrated that multiple clones may occur in CLL. Most therapy-related leukemias occur at 3 to 10 years after the initial therapy, with a longer latency for alkylating agents (5 to 9 years) [**1**].

Evolution to myelodysplastic syndrome or acute leukemia is rarely seen in patients with CLL, most reported cases have been therapy-related. Some authors hypothesize that these two diseases have evolved from a single pluripotent stem cell, while the others support the idea of the presence of two separate malignant clones [**11**].

Lima et al. demonstrated the presence of a common karyotype abnormality in both CLL (i.e., trisomy 12) and AML (i.e., monosomy 5, monosomy 7, and trisomy 21) lineages [**12**].

Alkylating-agent-related t-MDS/AML is associated with abnormalities involving chromosomes 5 (-5/del[5q]) and 7 (-7/del[7q]), and with a high frequency of multidrug resistance phenotype [**13**,**14**].

## Discussion

We reported this case because it is a complex one. We know that CLL patients are more exposed to develop a second neoplasm, especially after being treated with an alkylating agent, but the association CLL-MDS is an unusual one. Based on morphology and bone marrow biopsy, three years later, we established the MDS diagnosis. Then, the patient developed acute myeloid leukemia subtype 6, a very rare type of AML, which is considered to have a poor prognosis [**2**].

Our patient developed multilineage dysplasia three years after the initial therapy for CLL and then rapidly evolved towards acute leukemia, in just two months. Taking into account the rapid evolution of the patient’s disease we could not establish if the second malignancy was therapy related or the anemia from the first presentation of the patient in our clinic was the beginning of a myelodisplastic syndrome, which manifested especially after the immunosuppression induced by Alemtuzumab.

## Conclusion

Therapy related myeloid neoplasia is more aggressive, with a poorer prognosis than de novo myeloid neoplasia. We selected this case for presentation due to some particular features:

-our patient had poor prognostic factors for CLL-ZAP 70, lymphocytes doubling time

-the patient developed multilineage dysplasia three years after initial therapy for CLL

-the evolution to acyte leukemia was rapid, in only two months after developing multilineage dysplasia

-the patient developed acute myeloid leukemia subtype 6, a very rare type of AML
